# Case of Antral Abscess of Twenty Years’ Standing—Extraction of Root of Right Lateral Incisor—Recovery

**Published:** 1883-10

**Authors:** Thos. Bryant

**Affiliations:** Guy’s Hospital


### Case of Antral Abscess of Twenty Years' Standing—Extraction of Root of Right Lateral Incisor—Recovery.

UNDER THE CARE OF MR. THOS. BRYANT, F.R.C.S., GUY'S HOSPITAL.

The following instructive case was reported in the *Lancet* of July 21st. The amount of medical treatment the patient had received before her admission to the hospital is, very judiciously, not stated, but evidently she had previously sought relief, since we are told that the tumor "was lanced from time to time." We have no hesitation in saying that had the patient applied to a qualified dental practitioner she need not have suffered for as many months as she actually did years.—Ed.

Charlotte M——, aged thirty-two, a glovemaker, was admitted on May 13, 1881, into Lydia ward. Her parents are living.

Mother, brothers, and sisters healthy. Her father suffered from some urinary trouble. Twenty years ago the patient had a blow on the upper lip; the teeth became very tender, and two or three years later she noticed a swelling on the superior maxillary bone. This swelling increased for about five years, and had since remained pretty stationary. This swelling was lanced, and she derived much relief. It was afterwards lanced from time to time, but no cure had been effected.

On admission, the patient looked a healthy young woman. Her right upper jaw was much swollen, pushing forward the skin and dilating the right nostril. There had been no discharge from the nostril. The swelling bulged into the mouth, forming an enlargement about the size of a large walnut, situated in the anterior part of the roof. All the teeth had erupted. The eyeball on the affected side was not displaced. The mucous membrane of the right nostril was abundantly red, and there was a foul odor like ozæna. The nostril was quite free. Pressure on the teeth and alveolar process caused no pain, and gave no sensation to the fingers of crackling. The patient stated that when she was about fourteen or fifteen, and when her face was a little swollen owing to toothache, she had her right upper bicuspid tooth extracted. She had also lost her left upper bicuspid, and all her four second molars had decayed and broken away, the stumps only remaining. The stumps of the bicuspid teeth were also in the patient's head.

On May 24th the palate opening was enlarged, when the finger easily entered into a large cavity, evidently the antrum. The upper part was full of soft material, and some foul-smelling dark fluid escaped. On hooking the finger forwards towards the alveolus a sharp root of a tooth was discovered projecting into the cavity. This proved to be the root of the right second lateral incisor. This tooth was now extracted. The cavity was cleared out, and a sponge inserted. The extremity of the root was bare and discolored. The rest of the root was covered with a number of small, irregular, brownish excrescences resembling tartar. There was no membrane covering the tooth at all, and the root had evidently been bathed in pus for some time.

On June 5th the patient had learnt to wash out the cavity with solution of permanganate of potash. The discharge was almost *nil.* The naso-lateral fold had reappeared. The patient being almost well was discharged.—*The Journal of the Rritish Dental Association.*

